# Full-Mouth Rehabilitation of a Completely Edentulous Patient Using Four Axially Placed Implants per Arch With Delayed Loading: A Clinical Case Report With 12-Month Follow-Up

**DOI:** 10.7759/cureus.117481

**Published:** 2026-10-05

**Authors:** Shubham K Srivastava, Supriya Singh, Rabia Khan, Akshim Rana, Shilpi Asthana

**Affiliations:** 1 Department of Prosthodontics, Sardar Patel Post Graduate Institute of Dental and Medical Sciences, Lucknow, IND; 2 Department of Periodontology, Swami Vivekanand Subharti University, Meerut, IND

**Keywords:** delayed loading, dental implants, full-mouth rehabilitation, hybrid prosthesis, implant-supported prosthesis

## Abstract

Comprehensive full-mouth rehabilitation of patients with extensive tooth loss requires careful integration of implant distribution, prosthetic design, occlusal reconstruction, esthetics, phonetics, and biological considerations to achieve a stable and functional outcome. A 55-year-old female patient presented with extensive tooth loss associated with compromised mastication, esthetics, and oral function and was considered for comprehensive implant-supported prosthetic rehabilitation. A prosthetically oriented treatment strategy was formulated in which the anticipated definitive prosthesis and desired occlusal relationships were considered during implant planning and restorative sequencing. Four implants were strategically placed in an axial configuration in each arch, providing an alternative to the posterior tilted-implant configuration commonly associated with four-implant full-arch concepts. Despite adequate primary stability, a six-month delayed-loading protocol was adopted to permit osseointegration before definitive functional loading. The prosthodontic phase incorporated multi-unit abutments, splinted open-tray impression making, verification of the master cast using a rigid verification jig and one-screw test, passive framework assessment, controlled maxillomandibular registration, and individualized occlusal development before delivery of screw-retained hybrid prostheses. At 12 months, all eight implants remained clinically stable and functional, with satisfactory prosthetic retention, occlusion, esthetics, and patient-reported masticatory function, and without clinically evident biological or mechanical complications. The significance of this case lies in the integrated use of four axially positioned implants in each arch with delayed functional loading and a structured prosthodontic verification workflow for bimaxillary hybrid rehabilitation. Although a single case cannot establish comparative effectiveness or long-term predictability, it illustrates a prosthetically driven approach for carefully selected completely edentulous patients in whom axial implant placement is anatomically feasible.

## Introduction

Complete edentulism can significantly compromise masticatory efficiency, phonetics, facial esthetics, and oral health-related quality of life. Although conventional complete dentures remain an established treatment option, inadequate retention and stability, particularly in patients with residual ridge resorption, may limit their functional effectiveness. Implant-supported full-arch rehabilitation provides an alternative by improving prosthetic retention, stability, and patient satisfaction while facilitating the restoration of occlusal relationships and facial support [1-3]. However, successful rehabilitation requires a prosthetically driven treatment strategy that integrates residual bone availability, implant distribution, interarch relationships, restorative space, and the biomechanical requirements of the definitive prosthesis [4-5].

The rehabilitation of completely edentulous arches using four strategically distributed implants is an established treatment concept. The conventional All-on-4 approach incorporates anterior axial and posterior tilted implants to optimize anteroposterior implant distribution and has demonstrated favorable clinical outcomes [6]. Nevertheless, posterior implant tilting is not universally required, and an entirely axial configuration may be considered when the available alveolar bone and planned implant distribution provide appropriate support for the definitive prosthesis. Implant orientation and distribution should therefore be determined by individual anatomical conditions and prosthetic requirements rather than by a standardized surgical configuration. Furthermore, although immediate loading is widely employed in full-arch rehabilitation, delayed loading remains an established alternative that allows a healing period before definitive functional loading. The selection of an appropriate loading protocol should account for primary implant stability, bone quality, implant distribution, and the overall treatment objectives [7-8].

Screw-retained implant-supported hybrid prostheses offer a restorative solution for completely edentulous patients requiring comprehensive reconstruction of the dentition and associated soft-tissue contours. Their design facilitates the restoration of tooth position, lip support, phonetics, and occlusal relationships while providing prosthetic retrievability. Nevertheless, long-term functional success requires accurate transfer of implant positions, verification of the master cast, passive framework adaptation, appropriate occlusal development, and periodic maintenance to minimize biological and mechanical complications [9-11]. These considerations are particularly relevant when a limited number of implants support a rigid, full-arch prosthetic framework.

The clinical significance of the present case lies in the integration of an entirely axial four-implant configuration in both arches with a six-month delayed-loading protocol and a structured prosthodontic verification workflow. Unlike the conventional All-on-4 configuration, all eight implants were positioned axially within the available alveolar bone, with their distribution determined by the anatomical conditions and requirements of the definitive prostheses. Despite achieving primary insertion torque values of 30-35 Ncm, definitive functional loading was deferred for six months. The subsequent rehabilitation incorporated multi-unit abutments, splinted open-tray impressions, master-cast verification using a rigid verification jig and the one-screw test, passive framework assessment, controlled maxillomandibular registration, and screw-retained hybrid prosthetic reconstruction. Rather than introducing a new implant concept, this report illustrates the coordinated application of established surgical and prosthodontic principles in bimaxillary rehabilitation using a limited number of axially placed implants, with clinical and prosthetic outcomes documented over a 12-month follow-up period.

## Case presentation

A 55-year-old female patient reported to the Department of Prosthodontics with the chief complaints of difficulty in mastication, inadequate retention and stability of her existing complete dentures, and difficulty in speaking comfortably during function. The patient sought definitive rehabilitation to improve masticatory efficiency, prosthesis stability, phonetics, facial support, and overall oral function.

A detailed dental history revealed a history of progressive tooth loss over several years, primarily attributable to generalized chronic periodontitis. According to the patient, tooth mobility and recurrent periodontal problems had progressively affected multiple teeth, eventually necessitating sequential extraction of the remaining teeth. The patient reported that the loss of teeth had occurred gradually rather than as a consequence of a single traumatic event or acute dental condition. Following complete tooth loss, she was rehabilitated with conventional complete dentures. However, with prolonged use, she experienced progressively inadequate denture retention and stability, particularly during mastication and speech. She reported difficulty in chewing firm foods and occasional displacement of the dentures during functional activities. The patient also perceived a progressive deterioration in facial support and overall oral function following tooth loss.

The history of periodontal disease was associated with progressive loss of the natural dentition and subsequent residual ridge resorption. No history of maxillofacial trauma, congenital absence of teeth, or previous implant-supported rehabilitation was reported. The patient had not undergone previous implant placement or implant-related treatment. The principal functional consequences of complete edentulism included impaired masticatory efficiency, inadequate retention and stability of the conventional dentures, compromised speech during function, loss of dental and facial support, and reduction in lower facial height.

A comprehensive medical history was obtained to identify systemic conditions or medications that could influence surgical treatment, osseointegration, or prosthetic rehabilitation. The patient reported no known history of diabetes mellitus, hypertension, cardiovascular disease, bleeding disorders, osteoporosis, or other systemic conditions relevant to implant therapy. She was not receiving medications known to adversely influence bone metabolism or wound healing. There was no reported history of previous major surgical complications or adverse reactions to dental treatment. The medical history was therefore considered non-contributory to the planned implant-supported rehabilitation.

Extraoral examination revealed reduced lower facial height and diminished perioral support associated with complete tooth loss. The loss of dentoalveolar support resulted in a relatively collapsed lip profile and reduced support of the upper and lower lips. The facial profile, smile line, lip support, lower facial height, and overall facial symmetry were evaluated as part of the prosthetically driven treatment planning.

Intraoral examination confirmed complete edentulism of both the maxillary and mandibular arches. The residual ridges were assessed with respect to their morphology, height and width, mucosal condition, vestibular depth, interarch relationship, and available restorative space. Generalized resorption of the mandibular residual ridge was observed, whereas the maxillary ridge demonstrated relatively greater posterior resorption with associated reduction in available posterior bone volume. The mucosa was clinically suitable for implant-prosthetic rehabilitation, with no clinically evident pathological soft-tissue lesion identified at the time of examination.

The patient's previous experience with conventional complete dentures, together with progressive residual ridge resorption and persistent functional limitations, was considered insufficient to provide the level of retention and stability desired by the patient. Accordingly, implant-supported full-mouth rehabilitation was planned after comprehensive clinical and radiographic assessment. The history of progressive tooth loss secondary to generalized chronic periodontitis, combined with the subsequent limitations of conventional complete dentures, constituted the principal clinical background for the proposed implant-supported rehabilitation.

Diagnostic impressions and study casts were obtained to assess the residual ridge morphology, interarch relationship, and available restorative space. Panoramic radiography was used to evaluate vertical bone availability, relevant anatomical structures, and the proposed implant sites (Figure 1A). Radiographic assessment suggested satisfactory bone mineralization, based on the observed radiographic density, preserved cortical integrity, and relatively dense and well-organized trabecular architecture, with no apparent evidence of significant cortical thinning, porosity, or trabecular rarefaction [12]. Implant dimensions were selected based on a prosthetically driven assessment integrating the radiographic findings with clinical ridge morphology, mesiodistal site dimensions, available restorative space, and intraoperative assessment of the osteotomy and bone quality. Cone beam CT (CBCT) was not performed because no clinical or radiographic finding indicated a specific requirement for three-dimensional imaging; however, its absence is acknowledged as a limitation. Qualitative radiographic assessment demonstrated relatively reduced trabecular density and mineralization, particularly in the posterior maxillary regions, consistent with the reduced bone volume observed clinically. Cortical outline, trabecular pattern, and relative radiopacity were considered during treatment planning.

**Figure 1:**
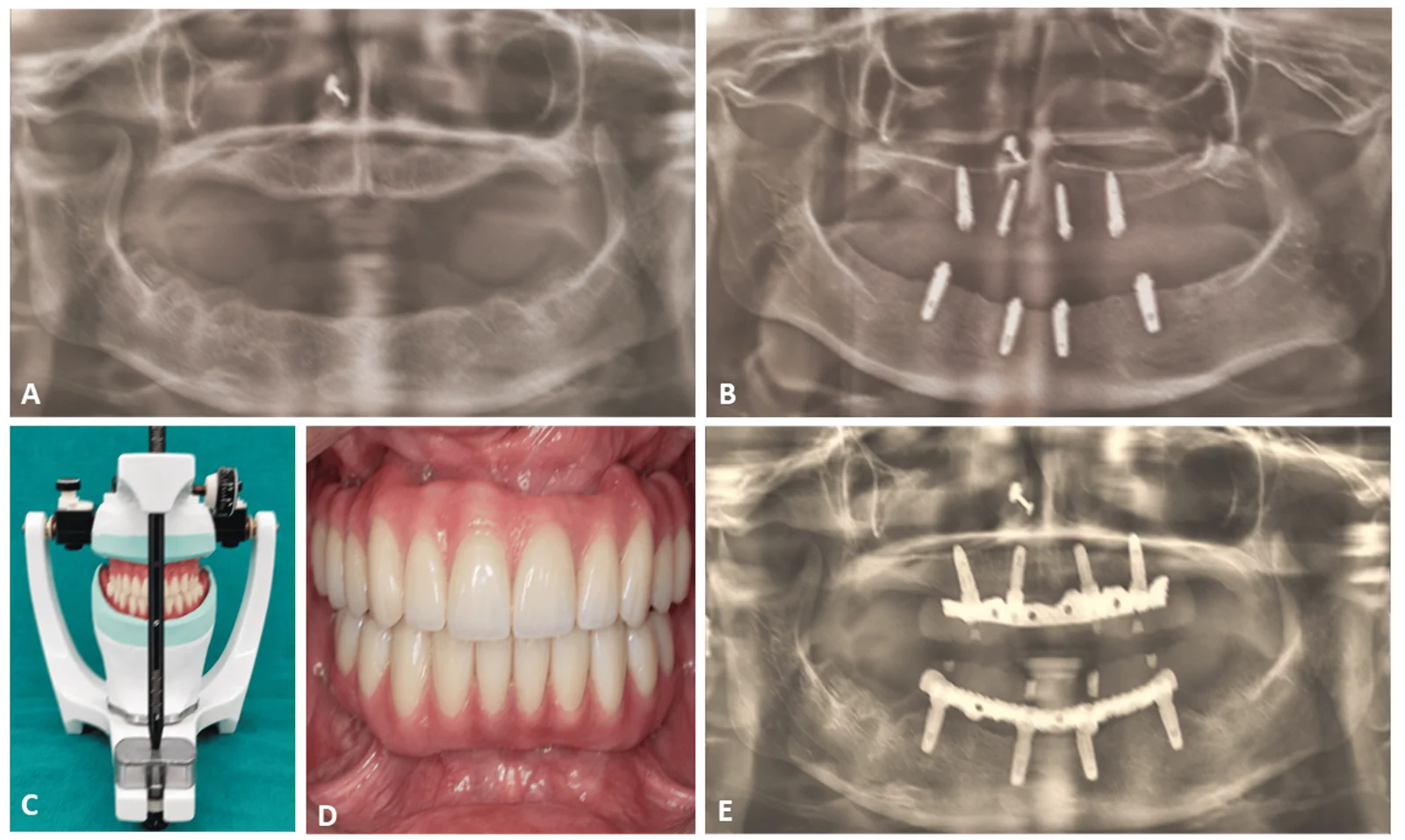
Clinical and radiographic sequence of full-arch implant-supported hybrid denture rehabilitation A: Preoperative panoramic radiograph demonstrating complete edentulism of the maxillary and mandibular arches with associated residual ridge resorption; B: panoramic radiograph demonstrating placement of four implants in the maxillary arch and four implants in the mandibular arch; C: maxillomandibular jaw relation and arrangement of prosthetic teeth for the hybrid denture on a semi-adjustable articulator; D: frontal intraoral view of the definitive implant-supported hybrid dentures in situ, demonstrating the anterior tooth arrangement and overall prosthetic integration; E: post-prosthetic panoramic radiograph demonstrating the definitive implant-supported hybrid prostheses and the prosthetic relationship of the implants following insertion.

Written informed consent was obtained from the patient for the proposed treatment and for the publication of the case report, including relevant clinical and radiographic images. Following comprehensive clinical and radiographic assessment, implant-supported full-mouth rehabilitation was planned for the completely edentulous maxillary and mandibular arches. A delayed-loading protocol was selected to permit adequate osseointegration before functional loading. Four endosseous implants (Osstem TS III SA, Osstem Implant Co., Ltd., Seoul, Republic of Korea) were planned in each arch and positioned axially within the available alveolar bone, with implant distribution determined by the residual ridge anatomy, restorative space, interarch relationship, and requirements of the definitive full-arch prosthesis. After administration of local anesthesia, a full-thickness flap was raised, and implant osteotomies were prepared using the implant surgical kit (Taper Kit Osstem, Osstem Implant Co., Ltd., Seoul, Republic of Korea) according to the manufacturer's recommended drilling sequence, and four implants were placed in the maxillary arch at positions (12, 15, 22, 25) and four in the mandibular arch (32, 35, 42, 45) with implants measuring 4.2 × 10 mm, 3.5 × 10 mm, 3.5 × 8 mm, and 4.2 × 8 mm, according to the anatomical characteristics of the respective implant sites, including the available bone dimensions and restorative requirements, followed by placement of cover screws (TS Cover Screw, Osstem Implant Co., Ltd., Seoul, Republic of Korea). An adequate antero-posterior distribution was achieved by positioning the posterior implants at the most distal anatomically feasible premolar sites (15, 25, 35, and 45) while maintaining axial orientation. Deliberate posterior tilting, as used in conventional All-on-4 protocols, was not considered necessary because sufficient bone was available at these sites for axial implant placement, and the resulting implant distribution provided satisfactory support for the planned full-arch prosthesis. The distal prosthetic extension was consequently controlled during framework design to minimize cantilever loading. Implant positioning and primary stability were assessed intraoperatively, and insertion torque values were recorded at the time of placement within the range of 30-35 Ncm. A mild angulation of the distal maxillary implants was accepted to accommodate the local residual ridge anatomy and available bone while maintaining the most favorable prosthetic trajectory. This angulation was anatomical and prosthetically guided rather than an intentional tilted-implant configuration and was selected to optimize implant positioning within the available bone while maintaining an appropriate path of prosthetic emergence. The surgical sites were sutured, and a postoperative panoramic radiograph was obtained to assess the position and angulation of the implants. Because a delayed-loading protocol was adopted, the implants were protected from definitive functional loading during the healing period. During the osseointegration period, the patient was provided with an interim removable complete denture, which was appropriately relieved over the implant sites and relined with a soft tissue-conditioning material (GC Tissue Conditioner, GC Dental Products Corp., Kasugai, Japan) to minimize pressure on the healing tissues and avoid direct loading of the implants. The patient was instructed regarding appropriate denture use, oral hygiene, dietary modification, and avoidance of excessive masticatory forces during the healing period.

After completion of the planned osseointegration period after six months, the implant sites were clinically reassessed to confirm satisfactory soft-tissue healing and implant stability, and second-stage surgery was performed to expose the implant platforms. Following removal of the cover screws, multi-unit abutments (TS Abutment, Osstem Implant Co., Ltd., Seoul, Republic of Korea) were connected to the implants according to the manufacturer's recommended torque specifications. Appropriate abutment height (2 mm) and angulation were selected (0° & 17°) based on the peri-implant soft-tissue thickness, implant orientation, available restorative space, and the desired common path of prosthesis insertion, and the protect cap (TS Protect Cap, Osstem Implant Co., Ltd., Seoul, Republic of Korea) was placed simultaneously for the peri-implant soft tissues to mature around the multi-unit abutments before initiation of the definitive prosthodontic procedures (Figure 1B).

To ensure accurate transfer of the three-dimensional implant relationship to the definitive master cast, open-tray impression copings were connected to the multi-unit abutments, and their complete seating was verified clinically, with radiographic confirmation when required. The copings were rigidly splinted using dental floss (Oral-B, Procter & Gamble, Cincinnati, OH, USA) and a low-shrinkage autopolymerizing pattern resin (GC Pattern Resin, GC Corporation, Tokyo, Japan), maintaining a minimal and uniform resin bulk to reduce polymerization shrinkage and heat generation. After polymerization, the resin splint was sectioned between adjacent copings and subsequently rejoined using a minimal amount of the same resin to compensate for polymerization-related dimensional changes and minimize internal stresses. A definitive open-tray impression was obtained at the multi-unit abutment level using polyvinyl siloxane (PVS) elastomeric impression material (Elite HD+, Zhermack S.p.A., Badia Polesine, Italy). Light-body PVS was syringed circumferentially around the impression copings and peri-implant soft-tissue margins, while the tray was loaded with the corresponding putty material and seated intraorally. After complete polymerization, the guide screws were loosened through the tray access openings, and the impression was removed carefully to maintain the positional stability of the impression copings. The corresponding multi-unit abutment analogs were then connected to the copings retained within the impression. A gingival replica material (Waldent FlexiGum Gingival Mask; Waldent/AlChem, India) was applied around the analogs to reproduce the peri-implant soft-tissue contours. The impression was subsequently boxed and poured with low-expansion type IV dental stone (Kalrock; Kalabhai Karson Pvt. Ltd., Mumbai, India) according to the manufacturer’s recommended protocol to obtain the definitive master cast. The accuracy of the master cast was subsequently verified intraorally using a rigid verification jig and the one-screw (Sheffield) test, in which the terminal screws were sequentially tightened to assess passive adaptation of the jig across the multi-unit abutments [13]. 

A facebow transfer was performed to record the spatial relationship of the maxillary arch relative to the cranial reference plane and facilitate accurate transfer of the maxillary cast to the articulator. The facebow record was transferred to a semi-adjustable articulator (Bio-Art A7 Plus; Bio-Art, São Carlos, Brazil), and the maxillary cast was mounted accordingly. The mandibular cast was subsequently mounted using the verified intermaxillary relationship at the predetermined occlusal vertical dimension. The centric relation record was repeated to confirm the reproducibility of the mandibular position before finalization of the articulator mounting.

Protrusive and bilateral lateral interocclusal records were obtained intraorally and transferred to the semi-adjustable articulator for individualized programming. The protrusive record was used to determine the sagittal condylar inclination, whereas the right- and left-lateral records were used to establish the corresponding Bennett angles. The articulator was subsequently adjusted to reproduce the recorded mandibular eccentric movements. The occlusal vertical dimension was established at 62 mm, with an interocclusal rest space of approximately 3 mm. Based on the recorded interocclusal relationships, the sagittal condylar inclination was set at 30° bilaterally, and the Bennett angle was adjusted to 16° bilaterally.

Once the master cast and maxillomandibular records had been verified, the definitive metal framework was designed to rigidly splint the implants through the multi-unit abutments while providing adequate restorative space for the prosthetic teeth and veneering resin. The framework contours were planned with consideration of implant distribution, framework rigidity, prosthetic space, hygiene access, peri-implant tissue contours, and control of the distal extension to minimize unfavorable mechanical loading. Following fabrication, the framework was clinically evaluated intraorally for complete seating and passive adaptation to the multi-unit abutments. Its stability was assessed for the absence of rocking, binding, or interfacial discrepancy, and the prosthetic screws were sequentially tightened to confirm consistent adaptation of the framework to the abutment interfaces. Definitive prosthetic processing was initiated only after satisfactory clinical verification of framework fit and passive adaptation.

The prefabricated prosthetic teeth were subsequently arranged in wax on the verified framework according to the established maxillomandibular relationship (Figure 1C). The wax trial was evaluated intraorally for the dental midline, tooth position, incisal display, lip support, occlusal plane, phonetics, and overall esthetic integration with the facial profile. The occlusal vertical dimension and maxillomandibular relationship were reassessed at this stage, and necessary modifications to tooth position and occlusal contacts were performed before definitive processing. Following clinical approval of the arrangement, the prosthesis was processed using the selected denture-base resin and prosthetic teeth. The processed prosthesis was subsequently finished and polished, with particular attention to the intaglio surface, polished contours, peri-implant hygiene access, and transition between the prosthetic flange and soft tissues.

The definitive prostheses were delivered as screw-retained, implant-supported hybrid prostheses and connected to the multi-unit abutments using the corresponding prosthetic screws (Figure 1D). The screws were tightened to the manufacturer's recommended torque using a calibrated torque-control device. The seating and adaptation of the definitive prostheses to the multi-unit abutments were initially assessed clinically, followed by radiographic verification to confirm complete seating and proper adaptation of the prosthetic components (Figure 1E). The screw-access channels were sealed using a light-cured resin-based composite restorative material (Neo Spectra ST; Dentsply Sirona, Charlotte, NC, USA). Occlusion was evaluated in maximum intercuspation and during eccentric mandibular movements, and selective occlusal adjustments were performed to achieve stable and harmonious contacts while minimizing premature contacts and excessive nonaxial forces on the implant-supported prostheses.

Following insertion of the definitive prostheses, the patient was enrolled in a structured maintenance and follow-up program, with clinical evaluations performed at one, three, six, and 12 months. At each visit, all implants were assessed for clinical stability and functional performance, while the implant-supported prostheses were evaluated for retention, stability, occlusal harmony, prosthetic integrity, and evidence of biological or mechanical complications. The peri-implant soft tissues were examined for signs of inflammation, and oral hygiene instructions were reinforced as required. Radiographic evaluation was performed periodically to monitor peri-implant crestal bone levels and the integrity and adaptation of the implant-prosthesis complex.

During the 12-month follow-up period, the patient demonstrated a favorable clinical course (Figures 2A-2B). At the 12-month evaluation, all implants remained clinically stable and functional, with no implant loss or clinically evident biological or mechanical complications. The definitive prostheses demonstrated satisfactory retention, stability, structural integrity, and occlusal function. Radiographic assessment revealed minimal peri-implant bone loss compared with the postoperative baseline. The patient reported satisfactory masticatory function, comfort, and esthetics and expressed overall satisfaction with the definitive rehabilitation. The preoperative complaints of difficulty in mastication and esthetic dissatisfaction were resolved following treatment. Overall, the rehabilitation demonstrated satisfactory functional, esthetic, prosthetic, and radiographic outcomes over the 12-month observation period.

**Figure 2:**
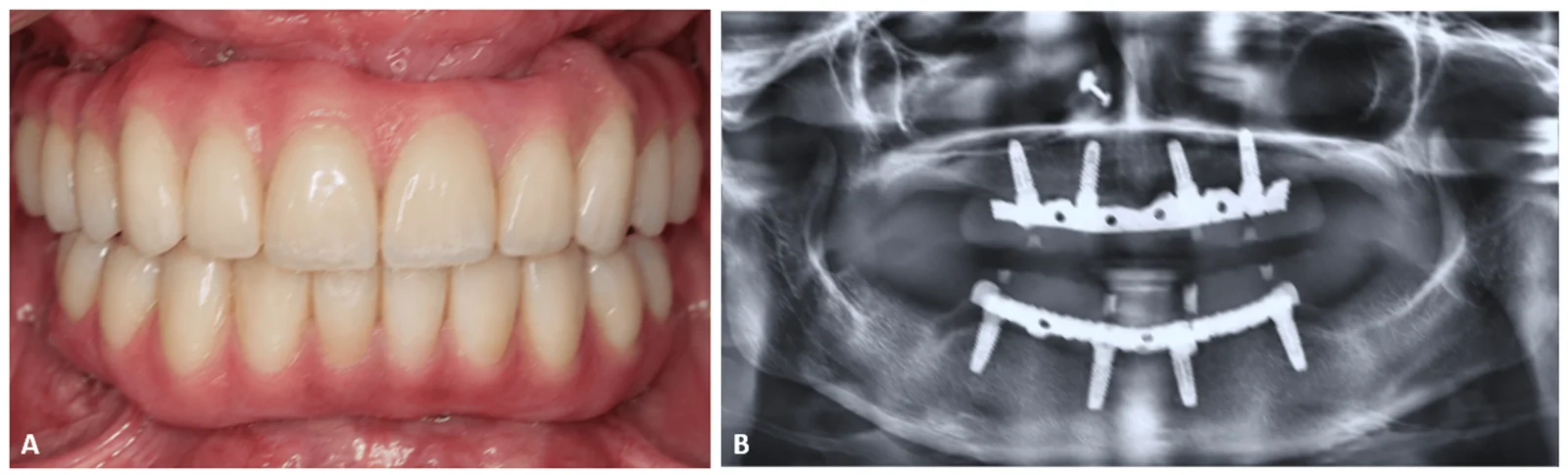
One-year clinical and radiographic follow-up A: Intraoral clinical photograph demonstrating stable full-arch implant-supported prostheses in occlusion, with satisfactory prosthetic appearance, maintained peri-implant soft-tissue health, and no clinically evident prosthetic complications; B: Panoramic radiograph demonstrating minimal crestal bone remodeling around the implants, with no apparent peri-implant radiolucency or other radiographic evidence of implant-related complications.

## Discussion

Complete edentulism is associated with impaired mastication, phonetics, facial support, and oral function, while conventional complete dentures may provide inadequate retention and stability in patients with advanced residual ridge resorption [1-3]. Implant-supported full-arch rehabilitation can improve prosthetic support and stability while facilitating restoration of the occlusal vertical dimension, facial support, phonetics, and esthetics. In the present case, the rehabilitation was performed using four axially positioned implants in each arch followed by a six-month healing period before definitive functional loading. The clinical significance of the case lies not in the use of four implants per arch alone, but in the coordinated application of an axial implant configuration, delayed functional loading, and a structured prosthodontic workflow for bimaxillary hybrid rehabilitation.

The use of four implants to support a fixed complete-arch prosthesis is an established treatment concept. The conventional All-on-4 protocol generally incorporates tilted posterior implants to increase the anteroposterior spread of implant support; however, four-implant rehabilitation does not inherently require posterior implant tilting. Comparative evidence indicates that four- and six-implant-supported fixed complete dentures can achieve favorable clinical outcomes when implant distribution and prosthetic design are appropriately planned [14]. A five-year multicenter randomized clinical trial reported non-inferior radiographic marginal bone-level outcomes for four implants compared with six implants in the edentulous maxilla, although technical complications occurred more frequently in the four-implant group [14]. Thus, implant number and configuration should be determined according to available anatomy, implant distribution, restorative space, and prosthetic requirements rather than by implant number alone.

A defining feature of the present case was the use of four predominantly axially positioned implants in each arch, with only mild anatomical angulation of the distal maxillary implants. The posterior implants were positioned at the most distal anatomically feasible sites while maintaining an axial orientation rather than intentionally reproducing the tilted posterior configuration of the conventional All-on-4 concept. A systematic review and meta-analysis reported predictable implant and prosthetic outcomes for both tilted and axially positioned implants, with lower marginal bone-level changes associated with axially positioned implants [15]. Nevertheless, implant orientation should remain anatomy- and prosthesis-dependent, and the present configuration was selected because the available bone and planned prosthetic design permitted axial implant placement without requiring deliberate posterior tilting.

The delayed-loading protocol was another important component of the treatment. The eight implants demonstrated insertion torque values of 30-35 Ncm, and definitive functional loading was postponed for six months to allow osseointegration before connection of the definitive prostheses. Evidence indicates that immediate, early, and conventional loading protocols can all achieve high implant and prosthesis survival in appropriately selected edentulous patients [16]. Therefore, immediate loading is not obligatory for successful full-arch rehabilitation, and delayed loading remains a clinically established option when the treatment plan favors a period of biological healing before definitive functional loading.

During the healing period, an interim removable complete denture was used with relief over the implant sites and a soft tissue-conditioning material. This arrangement maintained basic oral function and esthetics while minimizing direct pressure over the healing implants and supporting the intended delayed-loading protocol.

Accurate transfer of the intraoral implant relationship to the definitive working cast is critical in full-arch implant rehabilitation. In the present case, splinted open-tray impression copings, sectioning and rejoining of the splint, gingival-mask reproduction, and verification of the master cast were incorporated into the prosthodontic workflow. A review of implant impression techniques in completely edentulous patients reported greater accuracy with splinted than nonsplinted techniques and, in many investigations, with open-tray than closed-tray approaches [17]. These findings support the use of a splinted direct impression technique when multiple implants are required to support a common full-arch framework.

The verification jig provided an additional stage of clinical quality control before definitive framework fabrication. In the present case, the master cast was verified intraorally using a rigid verification jig and the one-screw Sheffield test to assess passive adaptation across the multi-unit abutments. A previous study reported a greater likelihood of achieving passive framework fit when a verification jig was incorporated into the fabrication sequence for nonsegmented implant-supported complete dentures [13]. This procedure is particularly relevant in rigid full-arch restorations because positional discrepancies introduced during impression making or cast fabrication may be transferred to the definitive framework.

Although minimizing framework misfit remains an important prosthodontic objective, the clinical consequences of a specific degree of misfit remain incompletely defined. A systematic review found insufficient evidence to establish a clinically acceptable threshold for prosthetic misfit and reported that greater discrepancies may be associated with screw-related complications, whereas a consistent adverse effect on peri-implant bone stability could not be demonstrated [18]. Accordingly, the objective in the present case was to achieve the most accurate and passive framework adaptation reasonably obtainable rather than to rely on a predefined numerical misfit threshold.

The subsequent use of facebow transfer, centric-relation verification, establishment of the occlusal vertical dimension, and individualized articulator programming provided controlled transfer of the maxillomandibular relationship to the laboratory phase. These procedures facilitated coordinated development of the definitive prosthesis and occlusal scheme. In the present case, occlusal contacts were adjusted in maximum intercuspation and eccentric movements to minimize premature contacts and excessive nonaxial loading while maintaining stable functional contacts.

The screw-retained hybrid prosthesis provided cross-arch implant support, retrievability, and flexibility in restoring tooth position, lip support, phonetics, esthetics, and occlusion. Long-term evidence demonstrates favorable survival of full-arch fixed hybrid prostheses and their supporting implants, although technical complications such as prosthetic tooth fracture or wear, acrylic-resin complications, and component-related complications remain clinically relevant [19]. More recent evidence similarly indicates that technical complications remain an important consideration during long-term maintenance of full-arch implant-supported prostheses [20]. These considerations emphasize the importance of prosthetic design, occlusal control, hygiene access, and structured maintenance.

At the 12-month follow-up, all eight implants remained clinically stable and functional, with no implant loss or clinically evident biological or mechanical complications. The definitive prostheses demonstrated satisfactory retention, stability, structural integrity, and occlusal function. Radiographic evaluation showed minimal peri-implant bone loss compared with the postoperative baseline, and the patient reported satisfactory masticatory function, comfort, and esthetics. These findings indicate a favorable short-term clinical course; however, they should be interpreted in the context of the single-patient design and limited follow-up duration.

This report has several limitations. A single case cannot establish comparative effectiveness or long-term predictability, and the absence of complications during 12 months cannot be extrapolated to longer periods of prosthetic service. In addition, CBCT was not performed, limiting three-dimensional assessment of buccolingual bone morphology and restricting extrapolation of the diagnostic approach to patients with more complex anatomical conditions. The favorable outcome should therefore be interpreted as a case-specific clinical observation rather than evidence of the superiority of axial four-implant rehabilitation over alternative full-arch protocols.

Overall, the present case illustrates the feasibility of combining four axially positioned implants in each arch with delayed functional loading and a structured prosthodontic workflow incorporating multi-unit abutments, splinted open-tray impressions, verification-jig assessment, passive framework verification, controlled maxillomandibular registration, and screw-retained hybrid prostheses. The 12-month outcome demonstrates satisfactory short-term functional, esthetic, and prosthetic results in a carefully selected completely edentulous patient. The principal clinical significance is the coordinated application of these established principles to achieve bimaxillary full-arch rehabilitation without relying on intentional posterior implant tilting.

## Conclusions

This case demonstrates the clinical feasibility of comprehensive full-mouth rehabilitation of a completely edentulous patient using four axially positioned implants in each arch, followed by a delayed-loading protocol and screw-retained hybrid prosthetic rehabilitation. A sequential prosthodontic workflow incorporating multi-unit abutments, splinted open-tray impression making, verification of the master cast, controlled maxillomandibular registration, passive framework adaptation, and systematic occlusal development facilitated the definitive rehabilitation. At the 12-month follow-up, all eight implants and the definitive prostheses remained clinically stable, with satisfactory functional, esthetic, and prosthetic outcomes and no clinically evident biological or mechanical complications. Within the limitations of a single case and the relatively short follow-up period, the findings illustrate that an individualized, prosthetically driven four-implant approach with delayed functional loading can provide a predictable short-term option for full-mouth rehabilitation in appropriately selected completely edentulous patients. Further long-term clinical studies are required to determine the durability and broader applicability of this treatment approach.
